# Synchronization of Acoustic Signals for Steganographic Transmission

**DOI:** 10.3390/s21103379

**Published:** 2021-05-12

**Authors:** Jarosław Wojtuń, Zbigniew Piotrowski

**Affiliations:** Faculty of Electronics, Military University of Technology, 00-908 Warsaw, Poland; zbigniew.piotrowski@wat.edu.pl

**Keywords:** audio steganography, audio watermarking, security, data hiding, synchronization

## Abstract

Steganography is a technique that makes it possible to hide additional information (payload) in the original signal (cover work). This paper focuses on hiding information in a speech signal. One of the major problems with steganographic systems is ensuring synchronization. The paper presents four new and effective mechanisms that allow achievement of synchronization on the receiving side. Three of the developed methods of synchronization operate directly on the acoustic signal, while the fourth method works in the higher layer, analyzing the structure of the decoded steganographic data stream. The results of the research concerning both the evaluation of signal quality and the effectiveness of synchronization are presented. The signal quality was assessed based on both objective and subjective methods. The conducted research confirmed the effectiveness of the developed methods of synchronization during the transmission of steganographic data in the VHF radio link and in the VoIP channel.

## 1. Introduction

The paper is devoted to the issues of acoustic steganography, and more precisely hidden synchronization of acoustic steganographic channels. Analyzing scientific publications in this field, it can be concluded that this is a valid topic, and the algorithms of acoustic steganography are constantly being improved. However, in many cases, the authors of the published solutions in their research ignore the significant problem of signal synchronization, in which data is embedded, often assuming perfect synchronization. In the case of practical implementations of steganographic systems, this approach is too much of a simplification, because achieving synchronization is a necessary condition for the effective extraction of payload [1,2].

Data transmission in a steganographic system is inextricably linked with the issue of synchronization. In the absence of synchronization mechanisms, the moment of starting the steganographic data extraction procedure is difficult to determine unequivocally, which implies the random nature of the received data, which is synonymous with low efficiency of hidden transmission.

The bit error rate (BER) was adopted as a measure of the efficiency of steganographic data transmission [3]. The use of hidden synchronization methods should therefore result in obtaining low BER values, which, in combination with detection and correction codes, will enable error-free transmission of the payload.

The use of hidden synchronization methods may or may not be associated with a deterioration in the quality of the cover work. Therefore, it is reasonable to search for such methods of synchronization that will not cause a significant deterioration of the quality of the signal carrying the payload. We often call cover work (original signal) with a payload Stego Object or Stego Work [1,2].

The paper presents four unique mechanisms that allow to achieve synchronization on the receiving side. Three of the developed methods of synchronization operate directly on the acoustic signal, while the fourth method works in the higher layer, analyzing the structure of the decoded steganographic data stream. All of new synchronization methods have been tested against the steganography paradigms: transparency, robustness, and data rate.

The remainder of the paper is organized as follows. Section 2 present a short description of the state of the art about speech steganography. Description of one of the methods of speech steganography is contained in Section 3. Technique development, implementation and study results are shown in Section 4 and Section 5, respectively. Finally, we summarize the paper.

## 2. Related Work

There are many published papers dealing with acoustic steganography. For the purposes of this paper, many solutions have been analyzed. The most popular and characteristic methods will be presented, which will sufficiently indicate how complex the problem is to hide information in an acoustic signal.

Depending on the place of embedding and extraction of payload in the speech signal in the telecommunications chain, the acoustic steganography algorithms can be divided into three groups [4].

The first variant consists of a certain modification of the operation of the selected speech signal codec. Such a mechanism was used, for example, in [5], where the G.729 codec code book was modified, which allowed for a hidden data rate of 2 kbit/s. In [6], it is proposed to hide information by changing the values of the linear prediction coefficients. The authors presented the results of experiments involving coding of the original signal with various codecs (G.721, GSM, G.728, G.729). In [7] the iSAC codec (internet Speech Audio Codec) was analyzed, hiding 12 bits per frame, which corresponds to 400 bit/s.

The second variant presents a situation in which hiding information is performed by modifying the data stream obtained at the output of the speech signal codec. The way of embedding information here is usually done by modifying the appropriate parameter or individual bits in the data stream. These types of solutions are relatively easy to implement and provide a high speed of payload transmission. Here we find a whole range of Least Significant Bits (LSB) methods from the simplest implementations [8,9,10,11] to the more complex [12,13,14].

The last, third variant involves embedding the information in the speech signal, just downstream of the analogue to digital converter, operating only on samples of the signal. The embedding of payload takes place with the use of various digital signal processing methods.

One way is to hide information by coding or manipulating the phase of the original signal [15,16,17,18,19]. Information hiding algorithms based on phase modification are characterized by high resistance to signal degrading factors and, depending on the carrier signal and the size of the data block being analyzed, by the hidden transmission rate from single bits to even kilobits per second [19,20]. A slightly different approach was proposed in [21]. Namely, instead of embedding the information in the phase of the original signal, the authors proposed to embed an OFDM signal in the original signal and encode the payload by changing the phase angle of these additional harmonics. This method is characterized by a data rate of about 40 bit/s and is resistant to degradation factors occurring in real VHF radio links or during speech signal transmission in GSM cellular lines.

In the works [17,22] a procedure of signal synchronization was presented, consisting in shifting, with a certain step, the receiving window in relation to the received signal and an attempt to extract the embedded bits. If periodically repeated maxima indicative of bit detection were obtained, the synchronization was considered to be achieved. In [20,21,23] the synchronization mechanism is described, the principle of which is based on the phase analysis of selected harmonics of the signal, in which the steganographic data is embedded.

Apart from the methods that modify the signal phase, there are methods that allow you to hide information by changing the amplitude spectrum of the signal [23,24,25,26,27,28]. These methods are characterized by a hidden data rate ranging from several bits per second to even several hundred bits per second. They are resistant to lossy compression, filtering, and changing the sampling frequency or analogue to digital conversion.

Apart from Fourier transform, often used in acoustic steganography, a number of publications are devoted to other transformations. In [29,30,31] the data hiding mechanism was presented, consisting in the quantization of the wavelet transform coefficients. In [29], a data rate of almost 300 kbit/s was achieved.

The method of using the cosine transform to hide data is described in [32]. The data rate of 150 bit/s was achieved, as well as resistance to lossy compression and analogue to digital conversion.

An innovative approach to the topic of hiding information in acoustic signals has been proposed in [33,34]. The authors propose to transform the acoustic signal into an image using the wavelet transform (A2IWT, Audio to Image Wavelet Transform). Then, embedding the information in the signal is done using one of the known steganography methods for digital images. The features of steganographic algorithms based on signal to image transformation depend on the properties of the image steganography algorithms used in a given case.

The paper [35] proposes a mechanism of steganographic data embedding in acoustic signals using the Hermit transform. The presented method is characterized by high perceptual transparency, but the authors do not specify the data rate achieved. The method is resistant to signal noise and filtering.

An algorithm based on the statistical properties of the signal was presented in [36]. In [37], the probability density function for the speech signal was proposed. Assuming that the speech signal at the receiver input is the sum of the steganographic signal and the noise signal, in [36] the dependencies on the random variable of this signal were determined. The process of embedding additional information in a single signal frame is done by appropriately scaling the amplitude. The data rate depends on the nature of the original signal and ranges from 172 bit/s for music signals to 40 bit/s for speech signals.

The papers [38,39,40] present the results confirming the use of the echo signal to create covert channels. These methods confirm effective data extraction in the presence of many signal distorting factors, such as adding noise, changing the sampling frequency, filtering, lossy compression, and transmission over a VoIP link.

In [41,42,43], methods using the spread spectrum were presented. Algorithms in the field are relatively easy to implement and show good resistance to a wide variety of signal transformations. They meet the requirements for perceptual transparency and provide a hidden data rate of several dozen bits per second.

There is, therefore, a relatively small number of articles and technical descriptions of audio systems used to create hidden communication channels. There is a clear gap in this area of knowledge. This is due in part to the realization that the power of the secret channel access key lies not in the number of key combinations but its stealth, i.e., the method of embedding and extracting the steganographic sequence. This is because the space of possible secret channel access key combinations is limited by strongly correlating with the values of the cover signal. The article [44] presents a mathematical description and explanation of this vulnerability.

The works on steganographic algorithms presented in this section do not exhaust this extensive issue. It is enough to bear in mind that in electronic publication databases, after entering the keyword audio steganography, only for the years 2018–2019 we get 92 results in the IEEE database, 121 results in the Web of Science database, and as many as 299 results in the Scopus database. On the other hand, the Google Scholar search engine finds 4270 items.

## 3. Embedding and Extraction Algorithm

In order to study synchronization methods, it is necessary to have a mechanism for embedding and extracting steganographic data. Among the many methods presented in Section 2, one of the algorithms was selected for further analysis. The algorithm is presented in [26,28], and described in detail in [45]. This method uses a narrowband speech signal as a carrier of steganographic data. Additionally, the authors showed that the method is resistant to a number of factors that degrade the steganographic signal during its transmission over the VoIP link.

For the purposes of this paper, there have been little changes introduced to the algorithm. The signal frame size was determined to be 192 samples (24 ms). Moreover, when determining the masking curve, a procedure using the psychoacoustic model of the MPEG-1 standard was used [46]. Hiding a single bit of information in a steganographic encoder consists of bipolar modification of the amplitude spectrum of the original signal in two adjacent signal frames. The steganographic data transmission rate was 20.83 bit/s.

The last modification of the original algorithm consisted in adding a feedback loop and a local decoder in the transmitting part, whose task is to constantly check whether it is possible to correctly extract the information bit embedded in the steganographic signal. In the steganography literature, such solutions are referred to as informed sender algorithms or “dirty paper codes” [47]. In case of error detection, a coefficient *C_i_* is determined at the output of the local decoder for the curve *SMR_i_(k)* (Equation (1)). In the feedback loop, we determine such value *C_i_*, for which the instantaneous signal value at the output of the local decoder, which is also the average *R* value of the previous instantaneous values, exceeds the specified threshold *K_min_*.

It should be additionally emphasized here that the greater the value of the signal at the output of the local decoder, the greater the energy of the watermark signal. Therefore, it will be more resistant to possible disturbances. At the same time, the higher energy value of the watermark signal makes it “audible” to the user of the system.

## 4. Technique Development and Implementation

The methods of signal synchronization in conjunction with the procedure of data embedding and extraction described in the Section 3 should allow for hidden data transmission in the selected telecommunications channel. The first method Monotonic Phase Correction and the second Direct Spread Spectrum of synchronization, consist of the construction of the synchronizing signal and adding it to the steganographic signal. The third method Pattern Insertion Detection consists of inserting a synchronizing marker into the speech signal preceding the steganographic transmission. The fourth and last method Minimal Error Synchronization, on the other hand, consists of the appropriate preparation of steganographic information.

### 4.1. Monotonic Phase Correction

**Figure 1:**
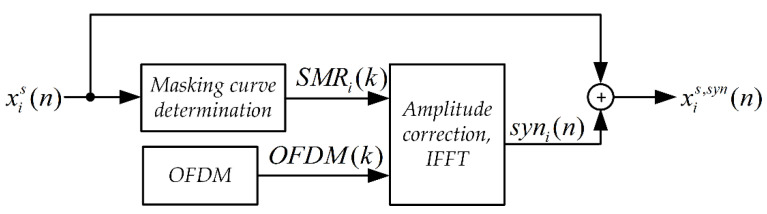
Block diagram of the synchronizing signal synthesis system for the MPC method.

The synchronizing signal synthesis system is shown in Figure 1. The input signal here is a Stego Object (signal in which steganographic information is embedded).

The *SMR(k)* masking curve was determined based on the psychoacoustic model of the MPEG-1 standard [46] according to the procedure described in [48]. For each frame and each harmonic component, the value of the correction factor was determined in accordance with the relationship.

(1) $$SMR_{i}(k)=SPL_{i}(k)-LT_{i}^{\min}(k)+C_{i},$$

where:

*i*—signal frame number,

*k*—harmonic number,

$SPL_{i}(k)$—sound pressure level for the *i*-th original signal frame,

$LT_{i}^{\min}$—the minimum masking threshold for the *i*-th original signal frame,

$C_{i}$—additional optional correction factor.

**Figure 2:**
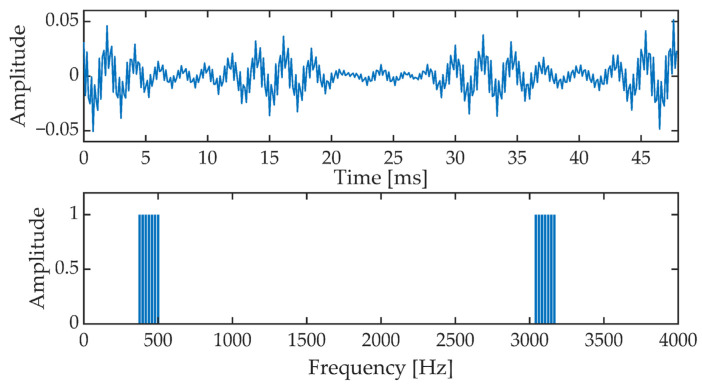
OFDM signal frame in time (upper picture) and frequency (amplitude spectrum, lower picture).

In an OFDM (Orthogonal Frequency Division Multiplexing) block, a signal is formed which is the sum of 14 harmonic components. The OFDM signal is contained in the band from 375 Hz to 500 Hz and from 3041.7 Hz to 3166.7 Hz. Figure 2 shows a single OFDM signal frame and the corresponding amplitude spectrum. The OFDM signal frame duration is 48 ms. The OFDM signal phase is set as follows:

(2) $$\varphi (k)=\{\begin{array}{c} 0,\;k=\left\{1,4,7,8,11,14\right\},\;\mathrm{pilots}\;\mathrm{harmonics}\\ \frac{\pi }{2},\;k=\left\{2,3,5,6,9,10,12,13\right\}\;\mathrm{synchronization}\;\mathrm{harmonics} \end{array},$$

**Figure 3:**
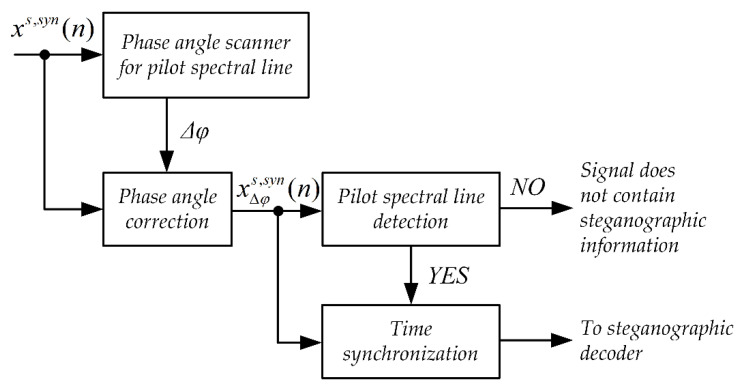
Block diagram of the synchronization system for the MPC method.

Figure 3 shows the synchronization system. The input signal $x^{s,syn}(n)$ is fed first to the input of the phase angle scanner system. The task of the phase angle scanner is to determine the value of the phase angle jitter [49,50]. This jitter may arise as a result of different accuracy of the clocks that clock the sampling circuits in the steganographic signal transmitter and receiver.

The next stage of the synchronizing system operation is the detection of pilot spectral lines. This procedure consists of checking whether a given pilot spectral line, after correcting its phase angle by the value of the determined jitter correction, has a phase angle value of zero. If the number of pilot spectral lines thus detected is greater than or equal to 4, then the input is assumed to be a steganographic signal and the algorithm moves to the timing step.

The time synchronization mechanism is based on the analysis of the cumulative phase of the signal. The cumulative phase is determined based on the recursive equation:

(3) $$\begin{array}{c} \chi_{1,k}=0\\ \chi_{i,k}=\chi_{i-1,k}+\varphi_{i-1,k}\;i\geq 2, \end{array}$$

where:

*i*—number of the analyzed signal frame,

*k*—harmonic number of the synchronization spectral line, the constant component has the index *k* = *0*,

$\varphi_{i,k}$—value of the phase angle of the *k*-th harmonic in the *i*-th frame.

**Figure 4:**
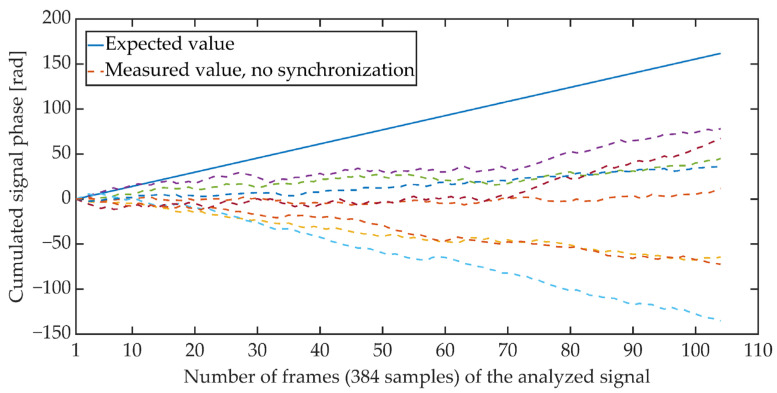
Cumulative signal phase, no synchronization.

**Figure 5:**
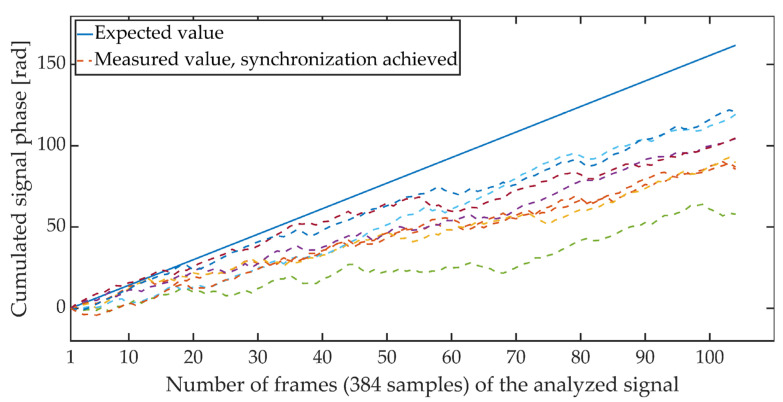
Cumulative signal phase, synchronization achieved.

Figure 4 and Figure 5 show the cumulative phase waveform for an exemplary steganographic signal, in which a synchronizing signal was additionally embedded. The continuous line marks the course of the cumulative phase of the signal on the transmitting side (in the synthesis circuit), and the dashed lines mark the courses of the cumulative phases recorded on the receiving side (in the synchronizer circuit). Figure 4 shows the cumulative waveforms of the signal in the absence of synchronization, and in Figure 5, when the synchronization is achieved. It is worth adding that for the presented characteristics, the average ratio of the steganographic signal energy to the energy of the synchronizing signal expressed in dB and determined in terms of segments (for 5 ms fragments) was 21.62 dB.

**Figure 6:**
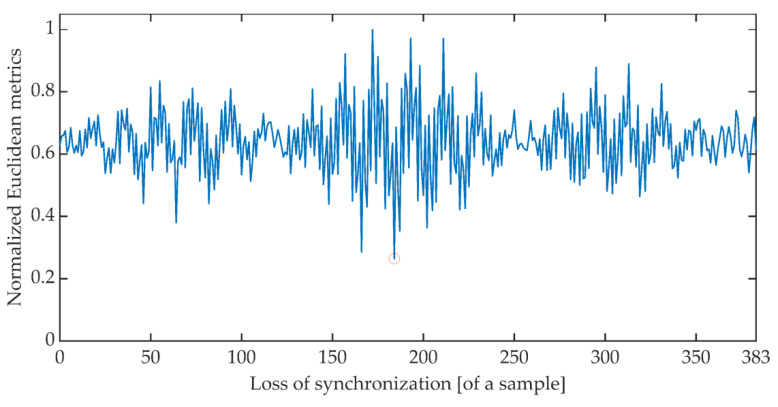
Cumulative normalized Euclidean distance between the expected cumulative phase and the measured phase.

**Figure 7:**
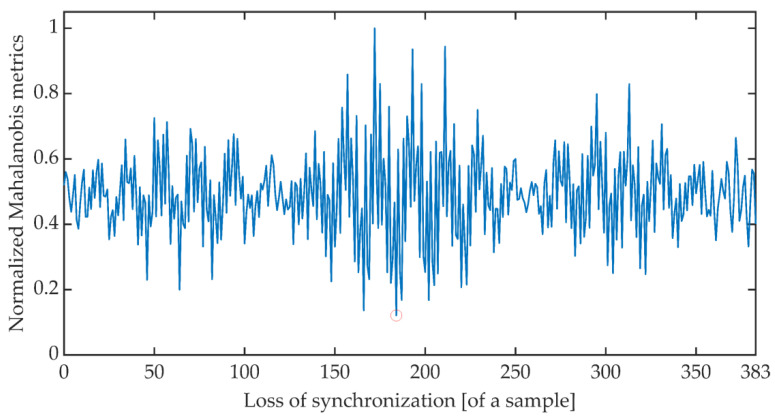
Cumulative normalized Mahalanobis distance between the expected cumulative phase and the measured phase.

**Figure 8:**
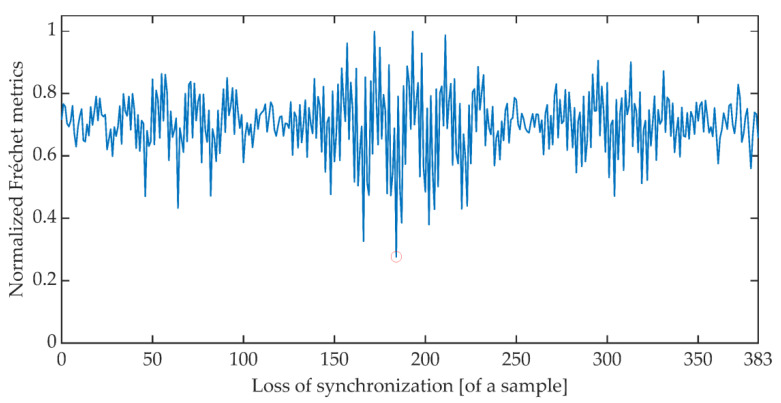
Cumulative normalized Fréchet distance between the expected cumulative phase and the measured phase.

The time synchronization procedure consists of an iterative search for such a signal detuning (shift) for which the distance between the expected value of the cumulative signal phase and the measured value is the smallest. Due to the periodicity of the OFDM signal, said minimum is searched for in the set of distances determined for offsets ranging from 0 to 383 samples. There are many different methods of determining the distance between data sets [51]. The work is limited to determining the synchronization using the Euclidean distance, Mahalanobis distance [52,53], and Fréchet distance [54,55]. Figure 6, Figure 7 and Figure 8 show the total distance between the expected cumulative phase of the signal and the phase measured using the above-mentioned metrics. Additionally, the figures show the minimum value of the determined distance. In the analyzed case, the minimum was achieved in each case for shifting the signal by 184 samples.

### 4.2. Direct Spread Spectrum

**Figure 9:**
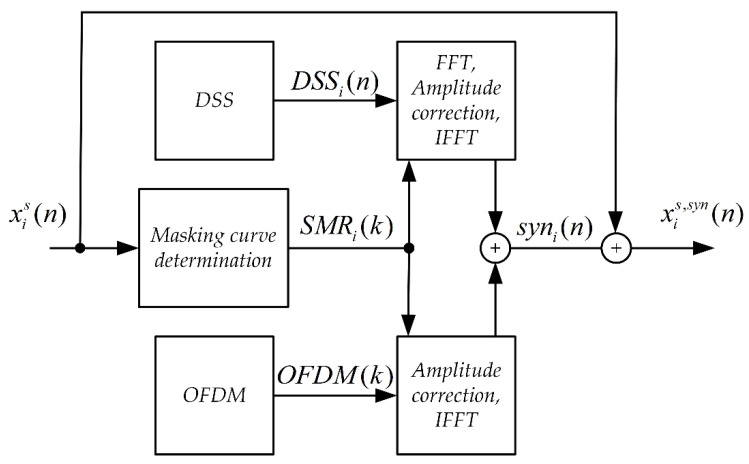
Block diagram of the synchronizing signal synthesis system for the DSS method.

The synchronizing signal generation circuit is shown in Figure 9. The input signal here is a Stego Object.

The *SMR(k)* masking curve was determined as described in the Monotonic Phase Correction method.

In an OFDM block, a signal is formed which is the sum of 6 harmonic components. The OFDM signal is contained in the band from 416.7 Hz to 500 Hz and from 3083.3 Hz to 3166.7 Hz. The OFDM signal frame duration is 24 ms. All harmonics of the OFDM signal act as pilot spectral lines. In the implementation, the value of the phase angle was assumed to be equal to 0.

**Figure 10:**
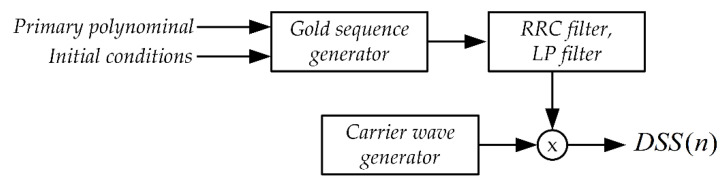
The block diagram of the DSS signal generation system.

The second component of the synchronization signal, next to the OFDM signal, is the DSS signal (Direct Spread Spectrum). The block scheme of the DSS signal generation system is shown in Figure 10.

The first stage of DSS signal synthesis is the generation of a pseudo-random sequence with appropriate properties [56]. Gold sequences and primary polynomials were used:

(4) $$\begin{array}{c} x^{13}+x^{9}+x^{7}+x^{5}+x^{3}+1={\left[8873\right]}_{DEC}\\ x^{13}+x^{9}+x^{5}+1={\left[8737\right]}_{DEC} \end{array},$$

with initial condition

[0000000000001]

The size of the Gold string used to generate the DSS signal has been limited to 6096 symbols. The duration of a single symbol has been set to *T_c_* = 1 ms. The duration of the entire sequence is therefore *T* = 6.096 s.

**Figure 11:**
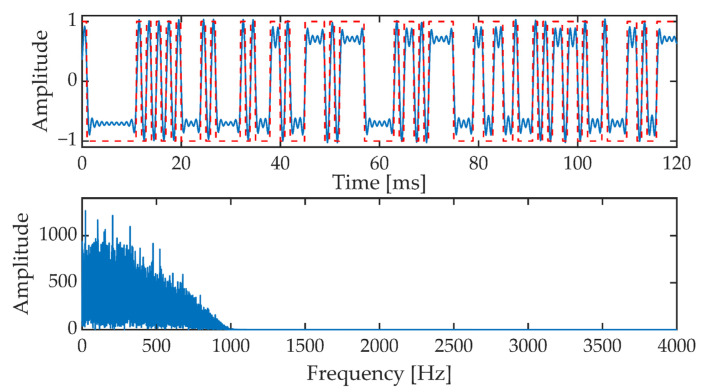
The signal at the output of the low-pass filter in the time domain (upper picture) and frequency domain (lower picture).

In the next stage, the generated pseudo-random sequence is fed to the input of the filter block. First of all, it is an interpolation filter with the characteristic of the root raised cosine (RRC, Root Raised Cosine) and then the low-pass filter such as FIR (Finite Impulse Response). These filters are designed to properly shape the pseudorandom sequence pulses and narrow the signal band. Figure 11 shows a fragment of the signal at the output of the low-pass filter. Additionally, the corresponding fragment of the pseudorandom sequence is marked (top picture, red dotted line).

The final step in generating the DSS signal is to transfer the signal from the low-pass filter output to a higher range of audio frequencies. This is due to the fact that frequencies below 300 Hz can be strongly suppressed during signal transmission in telecommunications links. The frequency of the carrier wave used is *f_c_* = 2000 Hz.

Spread spectrum systems are characterized by two important parameters processing gain *G* and the interference margin *M* [57]. The processing gain is a parameter that determines the degree of dispersion of the information signal spectrum:

(5) $$\begin{array}{c} G=\frac{T_{b}}{T_{c}}\\ G\left[dB\right]=10\log_{10}\left(\frac{T_{b}}{T_{c}}\right) \end{array},$$

where:

*T_b_*—duration of the data bit—synchronization bit,

*T_c_*—the duration of the spreading sequence chip.

Determining that the duration of the sync bit is equal to the duration of the spreading sequence *T_b_* = *T* = 6.096 s, the processing gain of the considered system is *G* = 37.85 dB. The obtained value of the processing profit meets the condition related to perceptual transparency.

The interference margin *M* is a measure of the receiver’s immunity to interference. It determines the maximum ratio of the noise power to the signal power at the receiver input, at which we obtain the minimum bit energy level to the noise power *E_b_/N*_0_ ensuring an acceptable error probability [56].

(6) $$M=G-\left(\frac{E_{b}}{N_{0}}\right)\left[dB\right],$$

After generating the DSS and OFDM signals, these signals are fed to the input of the amplitude correction circuit and then summed with the steganographic signal. Both the OFDM signal and the DSS signal are corrected based on the *SMR(k)* masking curve. At the same time, an additional condition is introduced for the DSS signal related to the correction of the signal energy. The correction factor *C_i_* (Equation (1)) is chosen such that for each signal frame the following condition is satisfied:

(7) $$10\log_{10}\frac{\sum_{n=0}^{N-1}{\left(x_{i}^{s}(n)\right)}^{2}}{\sum_{n=0}^{N-1}{\left(DSS_{i}(n)-x_{i}^{s}(n)\right)}^{2}}\leq M,$$

where:

$x_{i}^{s}(n)$—*i*-th frame of steganographic signal, noise signal for DSS signal,

$DSS_{i}(n)$—*i*-th frame of DSS signal.

In the receiving part, the synchronization procedure is based on a system similar to that shown in Figure 3. The difference is in the different principle of the time synchronization block.

**Figure 12:**
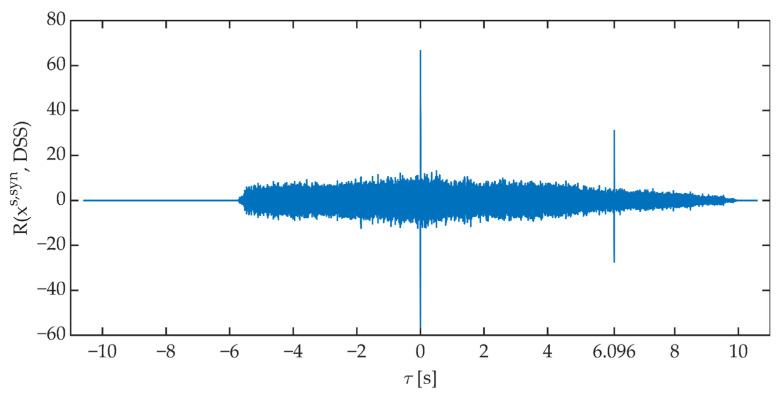
An exemplary course of the value of the cross-correlation function between the received signal and the DSS signal.

The time synchronization procedure consists of determining the value of the cross-correlation function between the received signal (corrected by the determined correction of the phase angle) and the reference signal generated on the receiving side. The received signal is “shifted” relative to the reference signal. The signals are considered synchronized when the value of the cross-correlation function reaches a maximum for *τ* = 0 s. Figure 12 shows an example of the course of the cross-correlation function value determined in the time synchronization block.

### 4.3. Pattern Insertion Detection

The synchronization method consists of inserting a synchronizing marker into the speech signal preceding the steganographic transmission. This method was developed for the needs of a steganographic system, which was designed to embed and extract data in a VoIP stream. 

The principle of operation is based on the analysis of the signal that will be the information carrier. The start of steganographic transmission is determined by the detection of the speech signal. The presence of speech in the signal is detected on the basis of the analysis of the values of two parameters [58]:

Signal power

(8) $$E_{i}=\frac{1}{N}\sum_{n=0}^{N-1}{\left(x_{i}(n)w(n)\right)}^{2},$$

ZCR (Zero Crossing Rate)

(9) $$ZCR_{i}=\frac{1}{N}\sum_{n=-\frac{N}{2}}^{\frac{N}{2}}\left|sign\left[x_{i}(n)\right]-sign\left[x_{i}(n-1)\right]\right|,$$

where:

$x_{i}(n)$—*i*-th frame of signal,

$w(n)=0.54-0.46\cos\left(\frac{2\pi n}{N-1}\right)$

*N*—number of samples in the signal frame.

**Figure 13:**
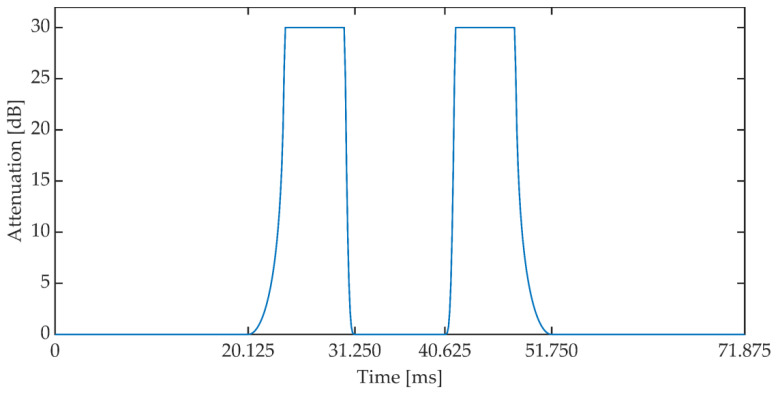
Signal attenuation pattern.

**Figure 14:**
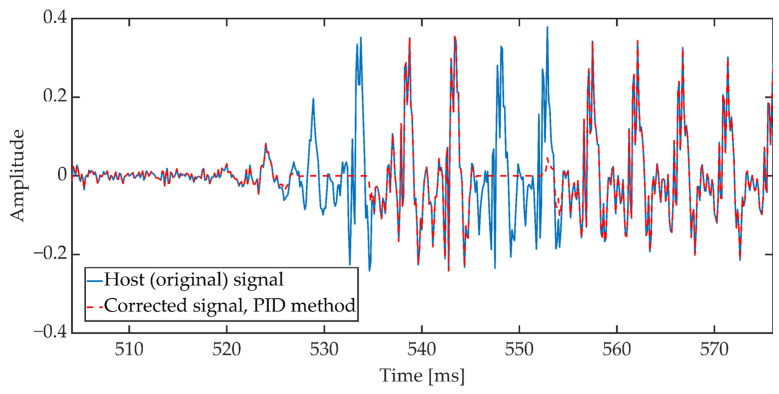
Sample waveform of the original signal and the signal corrected according to the attenuation pattern.

The duration of a single frame was set to 24 ms (192 samples). The implementation assumes that the presence of a speech signal is determined when the power value exceeds −50 dB and the number of zero crossings coefficient is less than 0.5. If three consecutive signal frames meet the above conditions, then these frames are corrected according to the attenuation pattern, the characteristics of which are shown in Figure 13. The characteristics of the attenuation pattern have been empirically established based on the preliminary research of the method. Document [59] states that the permissible IP (Internet Protocol) packet loss during the conversation should not exceed 3%. This value additionally depends on the speech signal codec used during communication. In addition, it assumes the use of the Packet Loss Concealment mechanism (PLC). The adopted attenuation pattern shape reduces the power of the speech signal in the 11 ms window, which is a value similar to the typical frame lengths in speech codecs used in VoIP. In three consecutive signal frames (72 ms) the mentioned reduction of the signal power occurs twice, see Figure 14.

The next stage, after performing the signal correction procedure (inserting a synchronizing marker into the signal), consists of embedding a portion of steganographic data in the speech signal, according to the algorithm described in the Section 3. The data portion size was set to 16 bits. The algorithm then restarts from scratch detecting the speech signal again.

In the receiving part, the synchronization procedure consists of continuously checking whether the currently analyzed signal fragment includes a synchronizing marker in its structure. This process is based on the analysis of the signal energy value and the number of zero crossings according to the Formula (8) and (9).

### 4.4. Minimal Error Synchronization

This method was developed for the needs of a steganographic system, which was designed to embed and extract data in a VoIP stream. The method was inspired by the cell delineation mechanism used in ATM (Asynchronous Transfer Mode) networks [60,61]. The purpose of the MES method is to recognize the steganographic transmission solely on the basis of the decoded bitstream, without the use of additional tags or unique sequences. The method of embedding and extraction of steganographic data remains unchanged as described in Section 3. It was assumed that the steganographic data extraction procedure would not know whether steganographic information was being transmitted at a given moment and that the extraction would always return a certain bitstream. Moreover, it was assumed that the steganographic data would be formed into a frame constructed in such a way that it would be possible to unambiguously recognize it in the bitstream after extraction, and that it would be resistant to 5% RTP packet loss.

The problem of recognizing a data structure in a bitstream is often solved by using a unique preamble or flag. However, in conditions of significant losses, and thus also distortions, such a mechanism cannot be used because it would generate incorrect frame recognition too often. Moreover, it is desirable that the data organization used should provide redundancy to repair bits corrupted due to RTP packet loss.

The transmission errors caused by the loss of RTP packets can be detected and corrected using detection and correction codes (Error Correction Code). There are many different variations of the code that can detect and correct errors. For the purposes of the paper, it was decided to use BCH codes (Bose–Chaudhuri–Hocquenghem). The choice of the BCH code was conditioned, on the one hand, by the requirement of the ability to improve the assumed percentage of lost RTP packets, and, on the other hand, by ensuring the lowest possible information overhead. In addition, the ease of implementation of the target steganographic system was of great importance here because the BCH encoding and decoding procedures are included in the Linux kernel.

BCH codes have strictly defined parameter values (*n*, *k*, *t*)

where:
*n* specifies the length (in the number of bits) of the code vector, $n=2^{m}-1$,
*m*—integer, *m* ≥ 3,
*k*—specifies the length (in the number of bits) of the information vector,
*t*—is the corrective ability of the code.

To determine the appropriate variant of the BCH code, which will enable the protection of steganographic transmission in the VoIP channel with RTP packet loss at the level of 5%, simulation tests were carried out. Two VoIP channel models were designed in the Matlab/Simulink environment:

- Model with PCMA codec;
- Model with iLBC codec variant 15.2 kbit/s.

The input signal was each time a speech signal with a duration of about 2 min, containing more than 2000 bits of payload. Packet losses were adjusted in the range from 0 to 5% with step 1. The payload was extracted on the receiving side. In the next step, the maximum number of errors recorded in a given observation window was determined. The observation window was shifted in the receiving vector every bit. Table 1 shows the maximum number of errors found in the receive vector with a size of *d* bits

Due to the specific values of the BCH codes parameters, the codes listed in Table 2 were selected for further analysis. In addition, this table shows the steganographic data rate *R* after taking into account the code rate and the minimum duration of the signal *T* to allow *n* bits of the code vector to be embedded in the signal.

The next stage of work on the method was to estimate the probability of the first type of errors. To this end, 10^7^ random bit sequences of length equal to n were generated for each variant of the BCH code, and then it was checked whether the BCH algorithm would qualify such a sequence as a BCH code vector. The results are presented in Table 3. For codes with the length of the code vector *n* = 63, the probability of the first type errors was considered too high. Two variants of the code with a length of *n* = 127 were selected for further analysis:

- *n* = 127, *k* = 50, *t* = 13;
- *n* = 127, *k* = 15, *t* = 27.

For the purposes of transmission, the code vector was interleaved.

In the receiving part, the synchronization procedure consists of continuously checking whether the BCH decoder can recognize the data frame in the extracted bitstream. If the BCH decoder determines that there are no errors or detects and corrects the errors, then it is assumed that synchronization is achieved. Otherwise, if the BCH decoder results in a negative syndrome, the speech signal is shifted by a certain number of samples and the steganographic data extraction and BCH decoding procedures are repeated.

It should be emphasized that the main disadvantage of the presented method is the high computational complexity related to the continuous operation of the steganographic decoder and the BCH decoder. On the other hand, it should also be noted that this is a method that does not interfere with the steganographic signal in any way. Therefore, there will be no deterioration in signal quality.

## 5. Results

### 5.1. Signal Quality Assessment

The methods of assessing the quality of audio signals can be divided into two main groups subjective and objective methods.

The objective assessment of the signal quality was carried out based on the ITU-T P.862 PESQ (Perceptual Evaluation of Speech Quality) [62]. Measurements were carried out using a dedicated MultiDSLA tester [63,64]. Documents ITU-T P.862 PESQ [62] and ITU-T P.862.3 [65] describe a number of requirements related to the selection of signals constituting the research material. The ITU-recommended test signals are contained in Annex B to ITU-T P.501 [66]. Samples for the Polish language were used in the research.

The MultiDSLA tester assesses the quality of the signals and returns the results in the form of several values:

- “raw” data (PESQ raw score or PESQ score);
- PESQ LQ (Listening Quality);
- P.862.1 (MOS—LQO, Mean Opinion Score Listening Quality Objective);
- P.862.2 (PESQ—WB).

Raw data changes range is from −0.5 to 4.5. These data are then transformed into the remaining results, the values of which range from 1 to 4.5 and correspond to the values on the MOS scale [67,68,69].

The subjective evaluation of the quality of the signals was based on the recommendations of ITU-R BS.1116-3 [70]. As with the objective tests, the document ITU-R BS.1116-3 describes a number of considerations on how to conduct the test. The study was conducted on a group of 20 students. A set of 16 test signals was used [71]. The test procedure consisted in listening by the listener of the original (undistorted) signal, marked as signal A, and two copies of this recording, marked as signals B and C, where, randomly, one of these signals is reference signal A and the other signal is distorted signal subject to evaluation. These types of tests are often referred to as “ABC (ABX) tests” or “forced choice tests”. The result of the assessment is the value in the SDG (Subjective Degradation Grades) scale, the range of possible scores is from −4 to 4. A positive value means that the listener has incorrectly determined which of the two signals he/she listens to is distorted.

**Figure 15:**
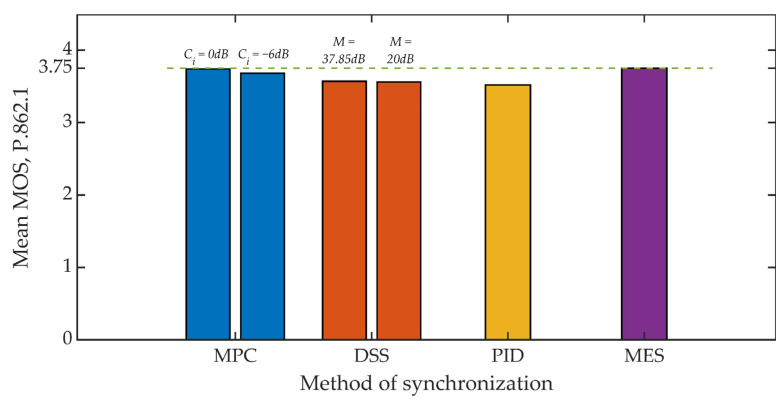
MOS evaluation for various synchronization methods, *K_min_* = 500.

**Figure 16:**
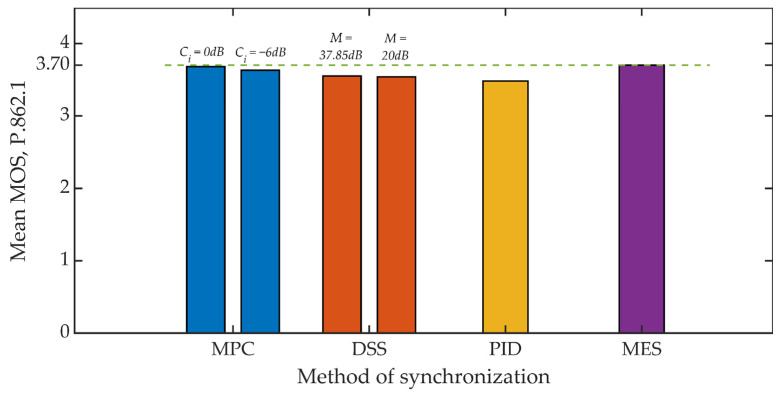
MOS evaluation for various synchronization methods, *K_min_* = 1250.

Figure 15 and Figure 16 show the results of the objective evaluation of signal quality carried out on the basis of ITU-T P.862 PESQ recommendations [62]. The line plotted in the drawings sets the reference level and represents the quality rating obtained by embedding steganographic information in the original signal. All of the developed methods, except for the MES method, reduce the evaluation of the signal quality. However, this is a slight reduction, amounting to about 6% for both analyzed values of the coefficient *K_min_*.

Summing up, it is worth adding that in all studies, the mean value of the MOS scale greater than 3 was obtained each time, which in the case of special applications, especially military, is a highly satisfactory result [72].

**Figure 17:**
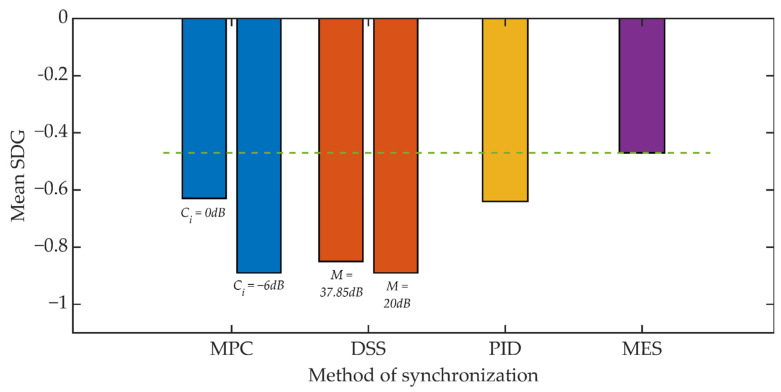
Listening test results for various synchronization methods, *K_min_* = 500.

**Figure 18:**
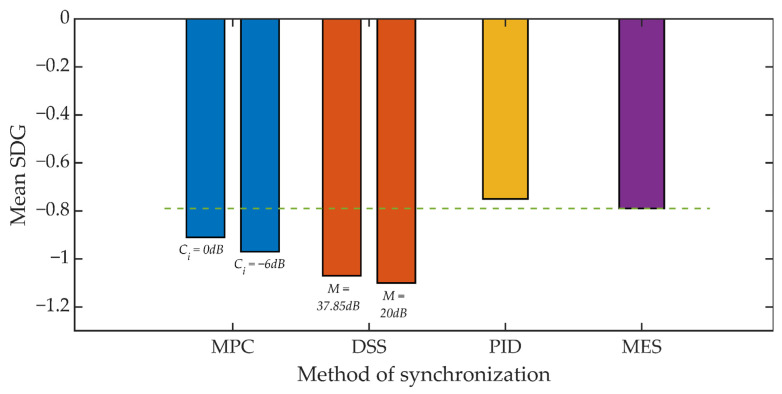
Listening test results for various synchronization methods, *K_min_* = 1250.

Figure 17 and Figure 18 show the results of the conducted listening tests. Again, the straight line defines the reference threshold and represents the SDG score obtained from tests that compared the original signal and a signal where only the steganographic information was embedded. This result is in line with the SDG assessment for the MES method. In the case of the value of the *K_min_* = 500 coefficient, individual methods of synchronization cause a deterioration of the SDG assessment in the range from 34% to 90% compared to the assessment for the MES method. However, it is worth noting that the determined average values do not take values lower than −1, which should be perceived as an inaudible signal distortion.

For *K_min_* = 1250 we can observe a much smaller, relative decrease in the SDG rating ranging from 15% to 40%. However, in this scenario, the benchmark is an SDG score of around −0.8. As a result, the SDG score for the DSS method slightly exceeds the value of −1.

### 5.2. Hidden Transmission Effectiveness Assessment

The aim of the study was to check whether the application of the developed mechanisms of synchronization of acoustic signals will allow for the implementation of steganographic transmission in a telecommunications channel in which there are signal degrading factors. The research was carried out using two different methods of steganographic signal transmission between the sender and the recipient. A teletransmission system based on radio waves and VoIP Internet telephony were used.

#### 5.2.1. Steganographic Transmission on the VHF Radio Link

**Figure 19:**
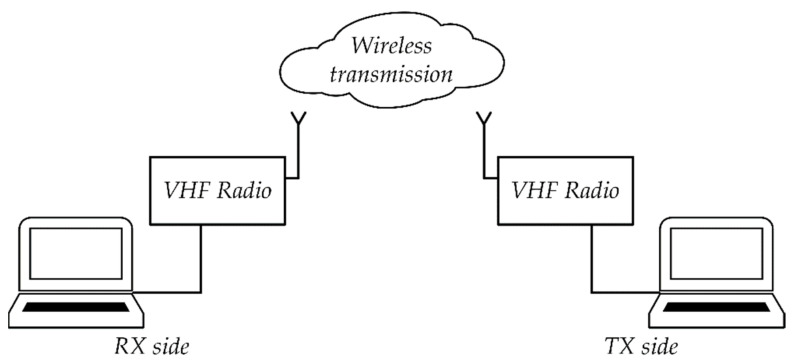
Diagram of a laboratory stand for testing steganographic transmission in the VHF radio link.

Figure 19 shows a laboratory stand for steganographic transmission. The stand was built on the basis of two computers and two RRC 9211 radio stations.

The research was carried out using three different radio modes:

- Analogue Fixed Frequency (AFF) with F3E;
- Digital Fixed Frequency (DFF) with F1D, CVSD 16 kbit/s and encryption;
- Fast Frequency Hopping (FFH) with F1D and CVSD 16 kbit/s and encryption.

The signals recorded on the receiving side were subjected to the synchronization procedure in accordance with the adopted synchronization method. Then, after obtaining the synchronization, the steganographic information was extracted. The bit error rate was adopted as a measure of the hidden transmission efficiency. The mean BER value and the 95% confidence interval (T-Student distribution) were determined. For the MES method, the BER value was determined based on the value of the BCH decoder syndrome. The study included one of the variants of the MES method for *k* = 50 and *t* = 13.

**Figure 20:**
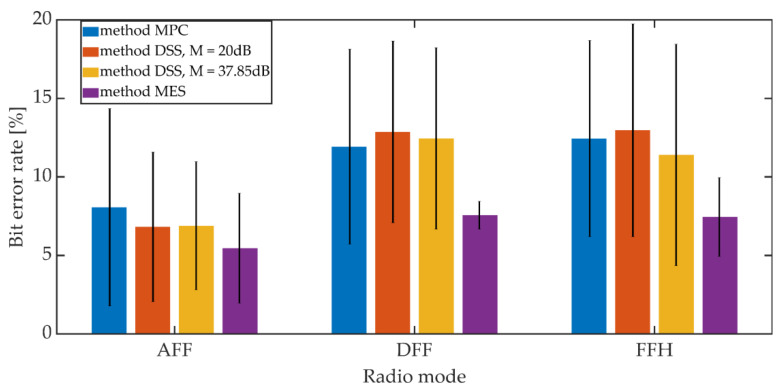
BER on the radio link for different synchronization methods, *K_min_* = 500.

**Figure 21:**
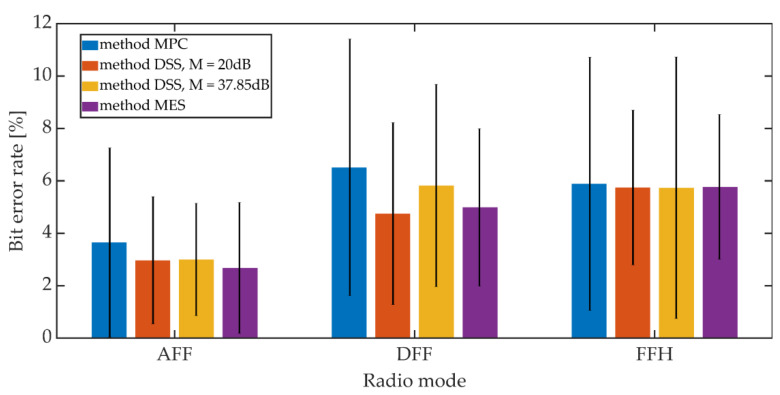
BER on the radio link for different synchronization methods, *K_min_* = 1250.

Additive noises occurring in the considered communication channel caused the distortion of the received signals. These distortions were so significant that they prevented the correct operation of the PID synchronization procedure. The obtained results are shown in Figure 20 and Figure 21.

Increasing the *K_min_* factor value from 500 to 1250 reduces the average BER value from 7% to 3% for AFF mode and from 12% to 6% for DFF and FFH modes. The higher BER values for digital modes may be due to the fact that in these operating modes there is lossy compression of the speech signal related to CVSD encoding.

Comparing the synchronization methods, we can see that the obtained bit error rate values in a given operating mode and for a fixed value of *K_min_* are similar to each other. Generally, the differences in values do not exceed 3.5% when analyzing both mean values and maximum values for the 95% confidence interval. 

#### 5.2.2. Steganographic Transmission in VoIP Channel

Steganographic transmission in the VoIP channel was made using the free and open source PJSIP library [73]. The application was compiled and run under the XUbuntu GNU/Linux operating system. The research was carried out for two variants of the network LAN and WAN.

The research was carried out using three different standards of speech signal coding:

- PCMA, 64 kbit/s;
- Speex, 24.6 kbit/s;
- G.729, 8 kbit/s.

The same test signals were used in the research that were used in the tests in the radio link [71].

**Figure 22:**
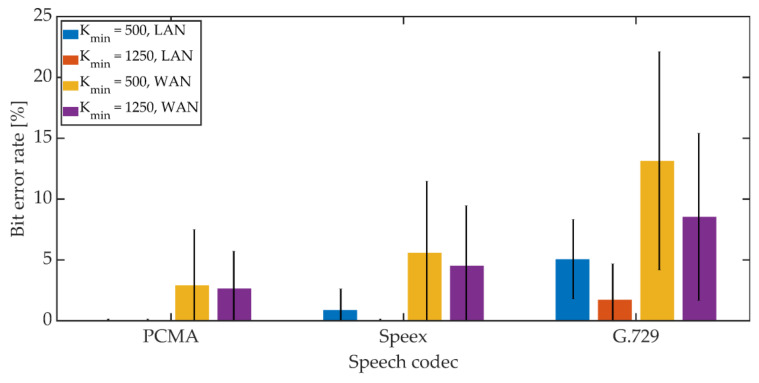
BER in VoIP channel for MPC method.

**Figure 23:**
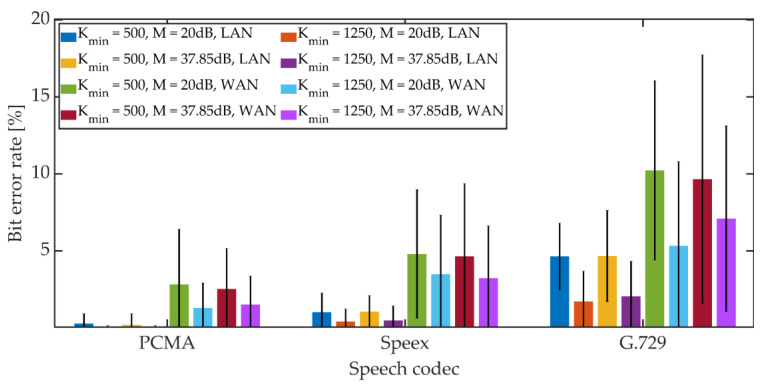
BER in VoIP channel for DSS method.

**Figure 24:**
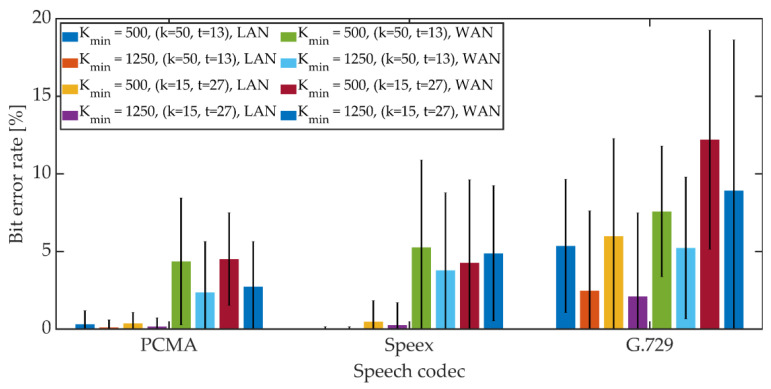
BER in VoIP channel for MES method.

The mean BER value and the 95% confidence interval (T-Student distribution) were determined. The obtained results are shown in Figure 22, Figure 23 and Figure 24.

For PCMA, the synchronization was achieved, allowing for the decoding of steganographic information for all methods. The use of Speex and G.729 codecs prevented synchronization in the PID method. As in the case of a radio link, an increase in the *K_min_* coefficient value from 500 to 1250 causes a decrease in the average BER value.

For PCMA and LAN, the BER value with the 95% confidence interval shall not exceed 1.2% for *K_min_* = 500 and 0.8% for *K_min_* = 1250. WAN transmission increases BER. The obtained values do not exceed 8.5% for *K_min_* = 500 and 6% for *K_min_* = 1250. Signal encoding using the Speex codec results in a BER level not exceeding 3% in the LAN and 12% and 10% in the WAN for *K_min_* = 500 and *K_min_* = 1250, respectively. Signal coding based on the G.729 codec translates into the largest number of errors during steganographic transmission in the VoIP channel. In the LAN, the BER value together with the 95% confidence interval does not exceed 9% (for MPC and DSS methods) or the number of errors is less than the BCH code correction capacity (for the MES method). Research in the WAN network allowed to obtain BER values below 16% for the MPC method and below 14% for DSS. In both cases, *K_min_* = 1250. In the case of the MES method for the BCH code with parameters (*k* = 50, *t* = 13), regardless of the value of the *K_min_* coefficient, problems with achieving synchronization were noticeable. Changing the coding variant to (*k* = 50, *t* = 27) significantly improved the efficiency of synchronization, and increasing the value of the *K_min_* coefficient to 1250 allowed for 100% synchronization efficiency.

## 6. Conclusions

The paper describes four new mechanisms that allow synchronization in acoustic steganography systems. All of these methods have been tested against transparency, robustness, and data rate.

The presented research results regarding the objective and subjective assessment of the quality of signals in relation to the developed methods of synchronization confirm the initial assumption that the use of hidden synchronization of acoustic signals will not significantly deteriorate the quality of the signal being the information carrier.

The presented research results on steganographic transmission in real telecommunications channels allow us to conclude that the use of hidden synchronization of acoustic signals increases the efficiency of steganographic data transmission in a telecommunications channel with signal degrading factors.

Machine learning algorithms can help increase the effectiveness of acoustic synchronization mechanisms. These algorithms build a mathematical model from sample data, called the training set. Machine learning that may prove helpful in the synchronization recovery process include the following methods: Decision Tree Learning for acquiring knowledge based on examples with numerous variants, Bayesian Learning as a probabilistic inference and Instance-based Learning method for modelling the synchronization procedure based on previous sample solutions. There are known methods of synchronization recovery for Forward Error Correction enabled channel [74] and the solution of the problem of network time synchronization [75] with the use of machine learning. Further work on the synchronization in acoustic steganographic channels should also cover the implementation of machine learning algorithms.

## Figures and Tables

**Table 1:** Maximum number of errors in the simulated VoIP channel.

Length of Observation Window *d*	PCMA	iLBC
31	7	6
63	7	8
127	7	10
255	10	15

**Table 2:** BCH code variants.

*n*	*k*	*t*	*R* [bit/s]	*T* [s]
31	6	7	4.03	1.488
63	18	10	5.95	3.024
63	16	11	5.29	3.024
63	10	13	3.31	3.024
63	7	15	2.31	3.024
127	57	11	9.35	6.096
127	50	13	8.20	6.096
127	43	14	7.05	6.096
127	36	15	5.91	6.096
127	29	21	4.76	6.096
127	22	23	3.61	6.096
127	15	27	2.46	6.096
127	8	31	1.31	6.096

**Table 3:** The probability of the first type of error.

*n*	*k*	*t*	*P*
31	6	7	1.06676·10^−1^
63	18	10	4.42610·10^−3^
63	16	11	5.43770·10^−3^
63	10	13	1.55420·10^−3^
63	7	15	2.40600·10^−3^
127	57	11	2.50000·10^−6^
127	50	13	1.10000·10^−6^
127	43	14	1.00000·10^−6^
127	36	15	1.00000·10^−6^
127	29	21	1.00000·10^−6^
127	22	23	1.00000·10^−6^
127	15	27	1.00000·10^−6^
127	8	31	4.00000·10^−7^

## Data Availability

The data presented in this study are available on request from the corresponding author. The data are not publicly available due to project restrictions.
